# Case of Fatal Hemorrhage from Perforation of the Arch of the Aorta, by False Teeth Impacted in the Œsophagus

**Published:** 1844-09-07

**Authors:** James Duncan

**Affiliations:** Surgeon to the Royal Infirmary of Edinburgh


### CASE OF FATAL HEMORRHAGE FROM PERFORATION OF THE ARCH OF THE AORTA, BY FALSE TEETH IMPACTED IN THE ŒSOPHAGUS.

By James Duncan, M. D. Surgeon to the Royal Infirmary of Edinburgh.

A young man, aged 22, dentist's workman, having lost his two front teeth by an accident, supplied the deficiency by a couple of artificial teeth secured on a frame in the usual way. He sometimes slept with these teeth in his mouth. On the 28th of February last, they were missing in the morning, and the young man complaining of pain and difficulty in swallowing, he applied to Mr. Syme, who with a probang detected a foreign body in the œsophagus, much beyond the reach of the ordinary forceps used for extracting foreign bodies from the gullet. The patient was removed to the Infirmary, and attempts were made to entangle the foreign body in a skein of thread connected with the probang, but in vain. By and by the pain subsided, and the patient left the hospital in the evening of the 9th day, March 8th. Next morning Dr. Duncan was hastily summoned to see him at home. In rising from bed, and crossing the room, he had become suddenly giddy, and had vomited a mouthful of blood. He was immediately removed to bed, and complained of a feeling of great weakness, and of some slight difficulty of breathing. His face was pale, and skin rather cold, but the pulse was of moderately good strength. From his description of what had taken place, I was lead to believe that the foreign body had been dislodged from its situation, and that it was possibly within reach of the forceps, with which I had provided myself. I accordingly requested him to sit up by the side of his bed, to enable me to make the necessary examination. This he did with ease, and without much assistance, expressing great anxiety to have something done to relieve him. The act of depressing the tongue, to enable me to introduce the forceps, produced vomiting, and a mouthful of dark fetid blood was discharged. This was immediately followed by another but much larger quantity of fluid of the same description, perhaps about eight or ten ounces, and the false teeth were heard to rattle against the vessel into which it was received. The patient was immediately aware of this, and his friends were overjoyed at what had taken place. Another mouthful of the same fluid was then ejected: an interval of a few seconds elapsed, and then a mouthful of bright arterial blood was discharged; a second, and a third followed, the lips became livid, the pulse at the wrist ceased, the patient gave one or two convulsive sobs, and expired.

An inspection of the body was readily obtained from the friends. The pharynx, the œsophagus, and stomach, along with the carotids, subclavians, and arch of the aorta, were removed entire, a ligature having been previously thrown around the duodenum to prevent the escape of the blood which had accumulated in the stomach, and to enable us to form an estimate of the quantity which had been lost. The œsophagus, stomach, and duodenum, were found distended with pretty bright arterial blood. The quantity could not be measured; but in the opinion of those present at the examination there could not have been less than eight or ten pounds. The pharynx and œsophagus were laid open by an incision posteriorly, carried as low as the cardiac orifice of the stomach. About 4½ inches from the rima glottidis there was an ulcerated perforation of the anterior part of the œsophagus, of about ⅜ths of an inch in length and three lines in breadth, passing obliquely upwards from the right to the left side. The edges of the perforation were rounded, and there was considerable surrounding injection of the mucous membrane. By this opening the probe could be readily passed into the aorta; but the latter vessel was not laid open at the time, it being thought better to immerse it for a day or two in spirits before doing so. On laying open the aorta subsequently, a perforation of about the size of a large crowquill was found about half an inch below the origin of the left subclavian artery. The opening was irregular in form, the edges everted, and at the lower part there was a pretty firm adherent coagulum. There was little or no vascular injection around this opening. The artery was otherwise perfectly healthy.—*London Med. Gaz.*

